# A Rare Presentation of a Hydatid Cyst in the Brain

**DOI:** 10.5334/jbsr.3983

**Published:** 2025-07-01

**Authors:** Kelly Di Dier, Sven Dekeyzer, Mania De Praeter

**Affiliations:** 1 UZ Ghent, Ghent, Belgium; 2 University hospital of Antwerp, Antwerp, Belgium

**Keywords:** Hydatid disease, Hydatid cyst, Intra-axial cyst, Echinococcus

## Abstract

*Teaching point:* When hydatid disease in the brain is suspected, thoraco-abdominal imaging is warranted to detect extracranial lesions.

When an uncomplicated intra-axial cyst is encountered, hydatid disease should be in differential diagnosis, especially in patients from or with travel history to endemic areas, or with known exposure to dogs, sheep or cattle. Thoraco-abdominal imaging may aid diagnosis as hydatid disease more commonly affects the liver and lungs and only rarely the brain.

## Case Report

A 14‑year‑old Turkish boy was presented to the emergency department with headache, nausea, and vomiting. Clinical examination revealed bilateral papilledema. Brain MRI was performed due to suspected intracranial hypertension.

**Figure 1:**
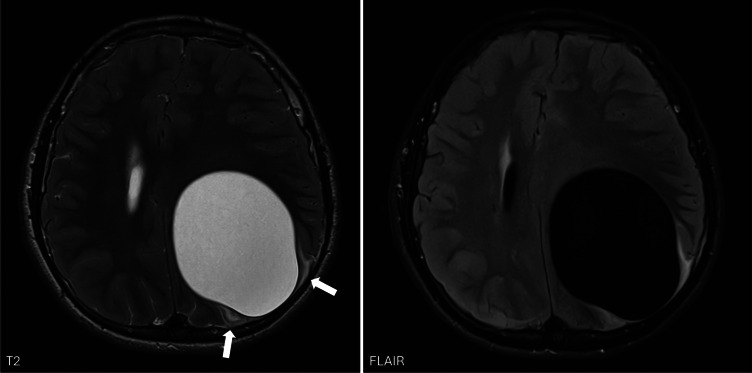
Axial T2‑ and FLAIR‑weighted images show a large intra‑axial cyst in the left frontoparietal region with signal intensity identical to cerebrospinal fluid. There is a significant mass effect with right‑sided midline shift but remarkably little surrounding edema (arrows).

**Figure 2:**
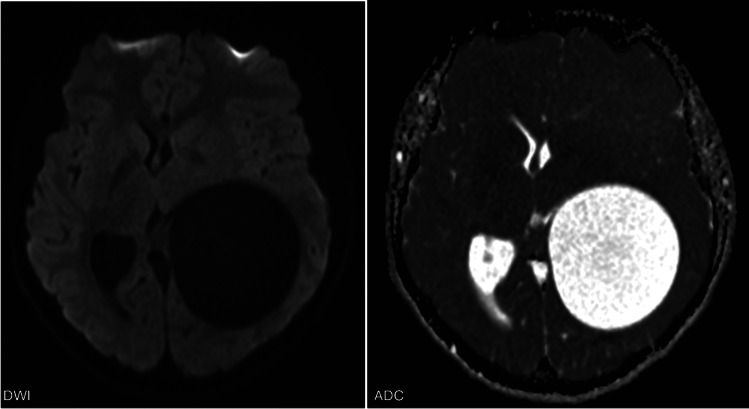
DWI images and ADC map show no diffusion restriction.

**Figure 3:**
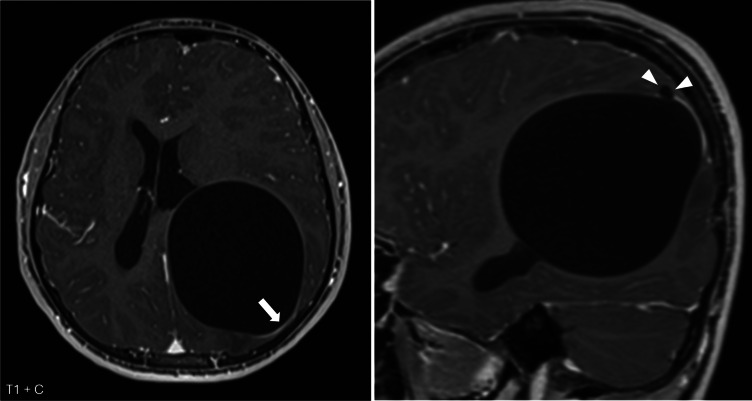
Axial and sagittal T1‑weighted images after contrast administration show slight rim enhancement at the parietal side (arrow), probably reactive to a focal breach in the cyst wall with the formation of a daughter cyst (arrowheads).

Imaging showed a large intra‑axial cyst in the left frontoparietal region with signal intensity identical to cerebrospinal fluid. The cyst exerted a significant mass effect with right‑sided midline shift but remarkably little surrounding edema (Figure 1, arrows)*.* There was no diffusion restriction (Figure 2) and only minimal rim enhancement, limited to a small area at the parietal side (Figure 3, arrows). This enhancement is believed to be reactive, secondary to a focal breach in the cyst wall with the formation of a daughter cyst (Figure 3, arrowheads).

Differential diagnosis included tumoral, congenital, and infectious cystic lesions. Cystic tumors often present with irregular rim enhancement or a mural‑enhancing nodule, neither of which was seen here. Congenital cysts, such as neuroglial cysts or porencephalic cysts, are not associated with mass effect. Infectious pyogenic brain abscess usually shows diffusion restriction, which was absent.

A parasitic origin was therefore strongly considered. Neurocysticercosis often presents as multiple intra‑axial cysts, not as a large solitary lesion. Hydatid disease was thus the most likely diagnosis.

**Figure 4:**
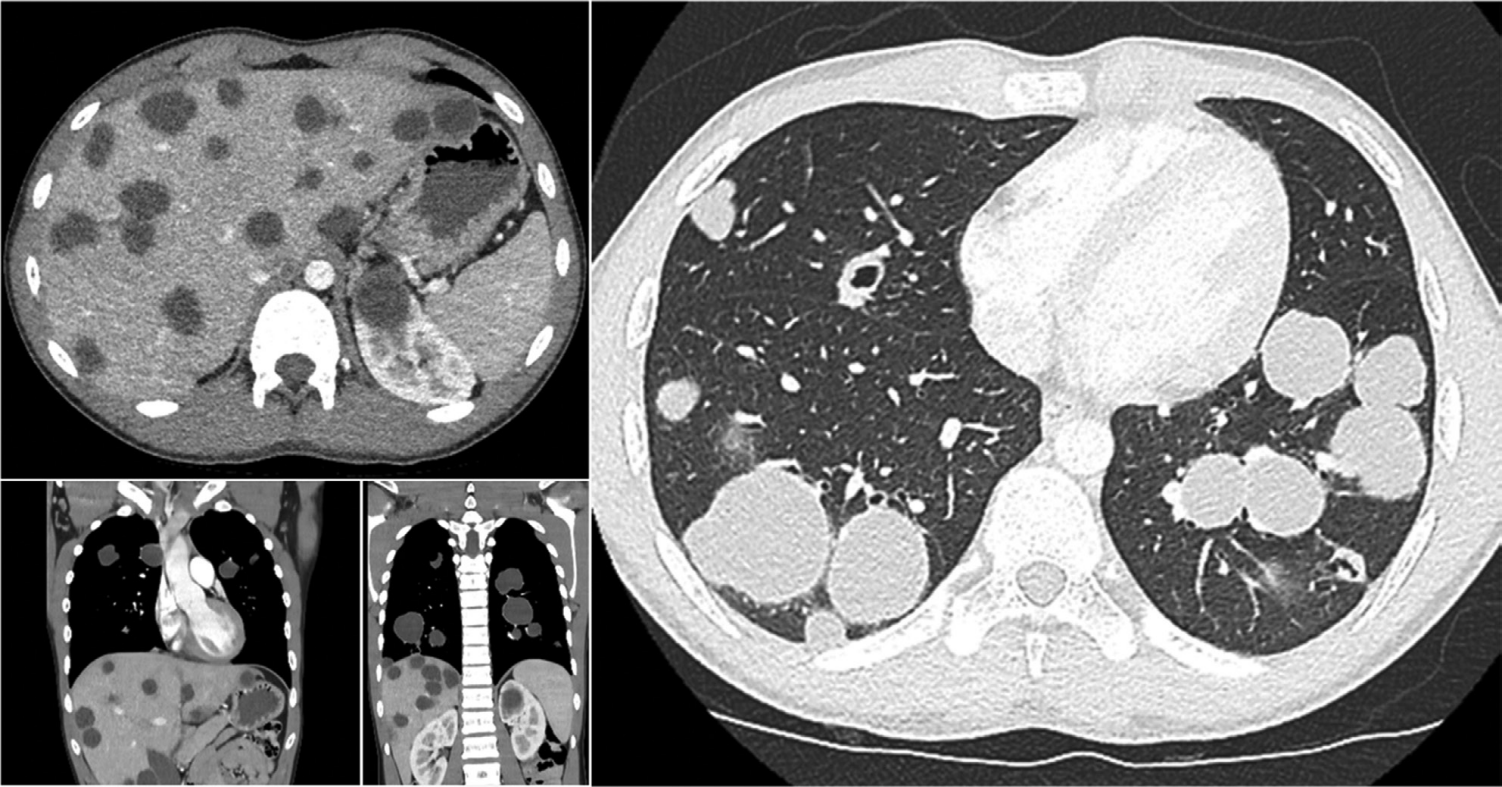
Axial CT images of the abdomen, coronal, and axial CT images of the thorax reveal plenty disseminated cystic lesions in the lungs and liver.

Given that hydatid disease more commonly affects the liver and lungs, a thoraco‑abdominal CT was performed. This revealed multiple cystic lesions in both lungs and liver (Figure 4), further supporting the diagnosis. The brain lesion was surgically removed, and the patient was treated with antihelminthic therapy. Complete resolution of symptoms was achieved.

## Comments

Hydatid disease is a zoonotic, parasitic infection caused by echinococcus tapeworms. It is endemic in Western China, Central Asia, South America, Mediterranean countries, and East Africa [1].

Hydatid disease is also known as cystic echinococcosis. The disease cycle involves definitive hosts (carnivores such as dogs and wolves) and intermediate hosts (herbivores such as sheep and cattle). The definitive hosts carry the mature tapeworm in their intestines and spread their eggs through their feces, contaminating water and food. Intermediate hosts ingest the eggs, which develop into cystic larval stages in internal organs. Humans can be accidental intermediate hosts. They acquire the disease through the same route as other intermediate hosts but do not contribute to the transmission cycle [1].

Once ingested, the eggs penetrate the intestinal wall and spread hematogenously via the portal system. The liver and lungs are the most commonly affected organs. Only 2–3% of cases involve the central nervous system [2].

Hydatid disease may remain asymptomatic for years. Symptoms depend on cyst location and size. Liver involvement can cause nausea, vomiting, and abdominal pain. Lung lesions can lead to coughing, chest pain, and dyspnea. Neurological symptoms vary based on lesion site [1].

Abdominal ultrasound is the modality of choice for hepatic involvement. CT complements abdominal imaging and is the first choice modality for pulmonary evaluation. Brain lesions can be detected with CT or MRI, although MRI is most sensitive [1].

There are no official guidelines for imaging in hydatid disease. Given the higher prevalence of hepatic and pulmonary involvement, thoraco‑abdominal imaging is warranted with a brain lesion that is suspicious for hydatid disease. Conversely, brain imaging may be considered in thoraco‑abdominal hydatid disease when neurological symptoms are present.

Treatment consists of antihelminthic therapy with surgical removal when indicated. Management depends on lesion size and symptomatology [1].
